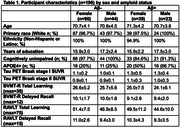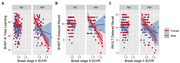# The moderating effects of sex and amyloid status on the associations between early tau burden and memory

**DOI:** 10.1002/alz.093224

**Published:** 2025-01-03

**Authors:** Kitty K. Lui, Xinran Wang, Jordan Stiver, Xin Wang, Erin E. Sundermann, Tobey J. Betthauser, Erin M. Jonaitis, Rebecca E. Langhough, Sterling C. Johnson, Sarah Banks

**Affiliations:** ^1^ SDSU / UC San Diego Joint Doctoral Program in Clinical Psychology, San Diego, CA USA; ^2^ University of California, San Diego, La Jolla, CA USA; ^3^ UC San Diego Health, La Jolla, CA USA; ^4^ Wisconsin Alzheimer’s Disease Research Center, School of Medicine and Public Health, University of Wisconsin‐Madison, Madison, WI USA; ^5^ Wisconsin Alzheimer’s Institute, University of Wisconsin‐Madison School of Medicine and Public Health, Madison, WI USA; ^6^ Wisconsin Alzheimer’s Disease Research Center, University of Wisconsin School of Medicine and Public Health, Madison, WI USA; ^7^ Wisconsin Alzheimer’s Disease Research Center, Madison, WI USA; ^8^ Wisconsin Alzheimer’s Institute, University of Wisconsin School of Medicine and Public Health, Madison, WI USA

## Abstract

**Background:**

Older women accumulate more pathological tau than older men in the early stages of Alzheimer’s disease (AD). However, the cognitive effects of early tau deposition may be obscured in women given their superior performance on verbal memory tests, which are commonly used in diagnosing AD. Visuospatial memory may more accurately reflect early tau burden in older women due to less apparent sex differences. In the Wisconsin Registry for Alzheimer’s Prevention (WRAP), we examined whether sex and Aβ status moderated associations between tau and verbal and visuospatial memory in older adults.

**Method:**

Cognitively unimpaired and mild cognitively impaired WRAP participants (n = 196; age = 70.8±4.2; 65.8% female) with [18‐F]MK6240 tau positron emission tomography (PET), [11‐C]Pittsburgh Compound B (PiB) Aβ‐PET, apolipoprotein E ε4 (*APOE4*) status, and memory scores from the Rey Auditory Verbal Learning Test (RAVLT) and Brief Visuospatial Memory Test‐Revised (BVMT‐R) were included (**Table 1**). Tau standard uptake value ratio (SUVR) in Braak stages I and II, regions of early tau deposition, were calculated. We used linear models to analyze the effect of sex×Aβ status (distribution volume ratio (DVR)>1.19)×tau interactions on memory, controlling for age at memory assessment, time between memory assessment and PET scans, *APOE4* status, and years of education.

**Result:**

Significant sex×Aβ status×tau in Braak stage II interactions were observed on BVMT‐R total learning, BVMT‐R delayed recall, and RAVLT delayed recall (*p*s<.03), showing that, in Aβ+ women, more tau in Braak stage II was associated with worse performance on these memory outcomes (*p*s<.01), but not in Aβ+ men (*p*s>.07; **Figure 1**). Moreover, in Aβ‐ men, more tau in Braak stage II was related to worse BVMT‐R delayed recall (*p* = .03), but not in Aβ‐ women (*p* = .97; **Figure 1B**).

**Conclusion:**

These findings indicate that, in the presence of Aβ pathology, there are impacts of early tau accumulation on both visuospatial and verbal memory in older women more than in older men. Longitudinal studies with ethnoculturally diverse samples will be necessary to clarify our understanding of the relationship between tau accumulation and memory decline and to increase the generalizability of the results.